# Multi-Component Biodegradable Materials Based on Water Kefir Grains and Yeast Biomasses: Effect of the Mixing Ratio on the Properties of the Films

**DOI:** 10.3390/polym15122594

**Published:** 2023-06-07

**Authors:** Agustina Lago, Juan F. Delgado, Guillermo D. Rezzani, Celeste Cottet, Yuly A. Ramírez Tapias, Mercedes A. Peltzer, Andrés G. Salvay

**Affiliations:** 1 Departamento de Ciencia y Tecnología, Universidad Nacional de Quilmes, Roque Sáenz Peña 352, Bernal B1876BXD, Argentina; lagoagui@gmail.com (A.L.); juan.delgado@unq.edu.ar (J.F.D.); guillermo.rezzani@unq.edu.ar (G.D.R.); celeste.cottet@unq.edu.ar (C.C.); yuly.tapias@unq.edu.ar (Y.A.R.T.); mercedes.peltzer@unq.edu.ar (M.A.P.); 2 Consejo Nacional de Investigaciones Científicas y Técnicas (CONICET), Godoy Cruz 2290, Ciudad Autónoma de Buenos Aires C1425FQB, Argentina; 3 Instituto de Tecnología en Polímeros y Nanotecnología (ITPN), CONICET-Universidad de Buenos Aires, Av. Las Heras 2214, Ciudad Autónoma de Buenos Aires C1127, Argentina; 4 Comisión de Investigaciones Científicas (CIC), Calle 526, La Plata B1900, Argentina

**Keywords:** biopolymer blend films, water kefir grains, yeast, biobased films, characterisation

## Abstract

The use of biopolymeric materials is restricted for some applications due to their deficient properties in comparison to synthetic polymers. Blending different biopolymers is an alternative approach to overcome these limitations. In this study, we developed new biopolymeric blend materials based on the entire biomasses of water kefir grains and yeast. Film-forming dispersions with varying ratios of water kefir to yeast (100/0, 75/25, 50/50 25/75 and 0/100) underwent ultrasonic homogenisation and thermal treatment, resulting in homogeneous dispersions with pseudoplastic behaviour and interaction between both biomasses. Films obtained by casting had a continuous microstructure without cracks or phase separation. Infrared spectroscopy revealed the interaction between the blend components, leading to a homogeneous matrix. As the water kefir content in the film increased, transparency, thermal stability, glass transition temperature and elongation at break also increased. The thermogravimetric analyses and the mechanical tests showed that the combination of water kefir and yeast biomasses resulted in stronger interpolymeric interactions compared to single biomass films. The ratio of the components did not drastically alter hydration and water transport. Our results revealed that blending water kefir grains and yeast biomasses enhanced thermal and mechanical properties. These studies provided evidence that the developed materials are suitable candidates for food packaging applications.

## 1. Introduction

The research and development of biobased materials for use in packaging is becoming increasingly important due to the current need for materials that can be obtained from renewable sources while also meeting requirements for processability and handleability [1,2,3]. In light of this, materials made from biopolymers, such as proteins and polysaccharides, are emerging as a viable alternative, including the biomasses from which these biopolymers originate [4]. Plasticisers are necessary to improve the integrity, flexibility and elasticity of biopolymeric films [2,5,6]. The most used plasticiser is glycerol due to its miscibility, biodegradability and its low cost [7].

While biopolymeric films have been considered promising for use in packaging applications, they often exhibit poor mechanical properties and thermal stability, which can negatively impact both processing and the final product [1,8]. To address these challenges, films made from blends of biopolymers can be designed to take advantage of the unique properties of each component, provided they are chemically compatible. This strategy can lead to materials with improved mechanical, thermal and barrier properties, as well as other functional characteristics [9,10]. Biopolymeric films and coatings can be used to cover food surfaces and act as barriers to control the transfer of moisture, oxygen, carbon dioxide, lipids and flavour components, maintaining the quality and increasing the shelf-life of food products [8,11]. In this approach, emphasis is placed on the biopolymers and the processes necessary to convert biomass components into materials with functional capacities comparable to those obtained from non-biodegradable synthetic polymers [1,4,12]. Among other important features, they can be used as carriers of functional agents, as antimicrobials or antioxidants [11,13] and to improve appearance and handleability [1].

The miscibility in polymer blends is assigned to specific interactions between polymeric components, which usually give rise to a negative free energy of mixing despite the high molecular weight of polymers [10]. Sometimes, two polymers are miscible in solution, but they are immiscible after solvent evaporation [10,14]. Blends of two or more synthetic polymers have been studied, but the blending of biopolymers is not well understood [14]. Some biopolymer blend films have been studied, such as starch/chitosan blend films [15,16,17,18,19,20,21], starch/whey protein blend films [22], chitosan/methylcellulose blend films [9], quinoa protein/chitosan blend films [23], kefiran/starch blend films [24], kefiran/chitosan blend films [25] and water kefir grains biomass/chitosan blend films [26]. These results suggested that the blending of biopolymers is a promising strategy to improve the properties of biobased films.

In recent years, new materials based on microbial biomass and its derivatives were developed [4], such as yeast biomass-based films [27] and water kefir grains biomass-based films [28]. Bakers’ yeast biomass is low cost and abundant with highly promising properties for the development of biodegradable materials because it is a natural blend of biopolymers; the composition of dry mass was previously determined as proteins (%): 41.2 ± 0.4; polysaccharides (mainly β-glucans and secondarily mannans) (%): 47 ± 1; RNA (%): 7.72 ± 0.04; and phospholipids and others (%): ~4 [29]. Yeast biomass-based films were developed in our laboratory and found to be homogeneous, continuous, compact, non-porous and non-crystalline [27]. The main components of yeast biomass, proteins and polysaccharides, can form a three-dimensional network without purification, resulting in a matrix that can be used to form a film if appropriate physical treatments are applied [27,30]. These films showed natural antioxidant activity, with a radical inhibition rate of around 50% [31], mainly due to the presence of β-glucans [32]. However, the colour characteristics, thermal degradation stability and mechanical properties of these films must be optimised for end-user applications [27].

The biomass from water kefir grains, which is obtained by fermenting a sweet and fruity aqueous medium [4], was used as the other component in the blends for this study. Water kefir grains consist of the exopolysaccharide dextran and contain a consortium of different micro-organisms responsible for the fermentation [33]. Dextran is a glucose polymer, mainly composed of linear α-D-1,6-linked with a low percentage of α-1,3-linked side chains [34]. Water kefir grain biomass-based films were also developed in our laboratory, showing excellent potential for film production [28]. Their transparency, thermal stability and mechanical properties for packaging applications were highlighted.

The natural components of yeast, such as proteins and glucans, possess unique characteristics that can be beneficial for food packaging. To capitalise on these properties and create a material with improved colourimetric, thermal and mechanical features, this study aimed to develop films using blends of the integral biomasses of yeast and water kefir grains. The goal was to investigate the effect of the mixing ratio of the biomasses on the functional properties of the films. Rheological properties of the film-forming dispersions were examined, and the resulting films were characterised through colourimetric, microstructural, spectroscopical, thermal, mechanical, hydration and water vapour barrier analyses.

## 2. Materials and Methods

### 2.1. Materials

Saccharomyces cerevisiae yeast pressed cells (Calsa-AB Mauri, Tucumán, Argentina) were purchased in a local market. Water kefir grains LOMCEM HWK1 were obtained from a household at El Pinar, Uruguay, and stored frozen at −20 °C. Dried Turkish figs, muscovado sugar and organic lemons were also purchased in a local market. Analytical grade salts used for the preparation of saturated solutions were acquired from Merck (Darmstadt, Germany). Analytical grade glycerol and silica gel were purchased from Biopack (Zárate, Argentina).

### 2.2. Preparation of Samples

#### 2.2.1. Water Kefir Grains Culture Conditions

Water kefir grains LOMCEM HWK1 were used as starter culture [28]. Frozen water kefir grains were reactivated by successive subculture at room temperature in a 2 L water cultivation medium containing ~100 g of kefir grains, 100 g of muscovado sugar, 50 g of dried Turkish figs and one lemon cut in four slices. The medium was exchanged every 48 h with a new culture medium. Subcultures were repeated several times to increase the production of water kefir grain biomass. In each subculture, the amount of biomass was doubled. To prepare the films, the grains were separated from the fermented product by filtering them through a plastic sieve. They were then washed three times with distilled water and pressed to remove the water excess. The amount of dry matter in washed and pressed kefir grains was 0.14 g per g, as determined by drying at 105 °C.

#### 2.2.2. Saccharomyces Cerevisiae Yeast Cells Conditioning

Before use, the commercial pressed bakers’ yeast cells were washed by dispersion in distilled water and centrifugation at 4 °C and 1000× *g* for 20 min. The supernatant was discarded, and the sedimented cells were used to prepare the film-forming dispersions. The washed yeast cell had a dry matter content of 0.22 g per g, as determined by drying at 105 °C.

#### 2.2.3. Preparation of Water Kefir Grains Dispersion

The washed and pressed kefir grains were used to prepare a dispersion containing 5 wt% dry matter (d.m.) in distilled water. To break down the structure of the grains, the dispersion was initially subjected to high-speed homogenisation at 15,000 rpm for 5 min using an Ultraturrax T-25 device (IKA Works, Inc., Staufen, Germany). Subsequently, it underwent ultrasonic homogenisation at 80 W via an Ultrasonic processor VCX-750 (Sonics and Materials, Inc., Newtown, CT, USA) for 15 min, following cycles of 30 s of pulse and 30 s of rest. The objective of the ultrasonic homogenisation process was to completely break down the grains and cell walls of the remaining micro-organism [28]. Thermal treatment in a water bath at 90 °C was then applied for 20 min to unfold biopolymers and deactivate enzymes and residual micro-organisms present in the media [28]. Finally, the dispersion was subjected to high-speed homogenisation at 15,000 rpm for 1 min to disassemble the possible aggregates formed by the thermal treatment.

#### 2.2.4. Preparation of Yeast Dispersion

Washed Saccharomyces cerevisiae yeast cells were used for the preparation of a dispersion containing 5 wt% d.m. of yeast using distilled water. The dispersion was first subjected to high-pressure homogenisation at 125 MPa and continuous flow for 9 min (High-pressure homogeniser NS1001L, Niro Soavi, Italy). This process was implemented to produce the rupture of the yeast cell walls and allow the release of the cytoplasmic content, mainly rich in proteins [29]. Then, thermal treatment in a water bath at 90 °C was carried out for 20 min to denature proteins and unfold other biopolymers, such as β-glucans and mannans [27]. Finally, the dispersion was subjected to high-speed homogenisation at 12,000 rpm for 1 min to break down residual aggregates produced by the thermal treatment.

#### 2.2.5. Preparation of Film-Forming Dispersions from Blends of Water Kefir Grains and Yeast Dispersions

Mixtures of the above-described dispersions of water kefir grains (K) and yeast (Y) were prepared in the following proportions: 100K/0Y, 75K/25Y, 50K/50Y, 25K/75Y and 0K/100Y. Ultrasonic homogenisation at 80 W for 15 min in cycles of 30 s of pulse and 30 s of rest was applied to produce fine dispersions. They were then vacuum-filtered using a Buchner funnel with filter paper, followed by complete degassing for 30 min using a vacuum pump. Finally, pure glycerol was added to dispersions at a level of 25 wt% with respect to d.m., and stirring was applied for 15 min. The final pH of the film-forming dispersions 100K/0Y, 75K/25Y, 50K/50Y, 25K/75Y and 0K/100Y were 6.35, 6.19, 5.40, 5.49 and 4.13, respectively.

To obtain films with thicknesses close to 0.13 mm, 20 g of the film-forming dispersion was placed in each plastic Petri dish, which was 86 mm in diameter. Water evaporation was carried out at 37 °C and 40% relative humidity (r.h.) by casting in a ventilated oven (Sanyo MOV 212F, Japan) until the remaining water content of the films was between 10 and 15%. This process took approximately 12 h. The films were then stored at 22 °C and 43% r.h. Finally, to meet the experimental requirements, the films were equilibrated at 22 °C in desiccators at different r.h. using saturated solutions of NaOH, MgCl_2_, K_2_CO_3_, Mg(NO_3_)_2_, NaBr, NaCl and BaCl_2_ to generate conditions of 10, 33, 43, 52, 57, 75 and 90% r.h., respectively. Dried atmospheres were achieved using silica gel.

### 2.3. Rotational Rheology Measurements of Film-Forming Dispersions

Flow curves were obtained through rotational tests using an AR-G2 rheometer (TA Instruments, New Castle, DE, USA) equipped with a 2° cone geometry steel plate (40 mm in diameter and 55 μm of gap space). Measurements were performed in triplicate at 22 °C using 1 mL of film-forming dispersions and increasing shear rates (0.01 to 125 s^−1^). Shear stress *τ* (Pa) as a function of shear rate *γ* (s^−1^) was measured, and the curves were fitted using the Herschel–Bulkley model shown in Equation (1):

(1) $$\tau =\tau_{0}+K\;\gamma^{n}$$

where *τ*_0_ (Pa) is the yield stress, which represents the maximum value of *τ* for a strain rate equal to zero; *K* is the fluid consistency index; and *n* is the flow behaviour index indicating the deviation to Newtonian flow type (*n* > 1 dilatant and *n* < 1 pseudoplastic).

### 2.4. Characterisation of Films

#### 2.4.1. Thickness Measurement and Quality Evaluation

Film thickness was measured using a digital calliper (±10^−6^ m; 3109-25-E, Insize Co., Suzhou, China). Measurements were taken at twenty different locations on the films, and for each specimen, an average value of 0.13 mm was obtained with an error of less than 5%. The quality of the films was assessed manually and visually based on their handleability, homogeneity and continuity [35]. Three independent replicates were conducted.

#### 2.4.2. Colour Determination (CIELab Coordinates)

The colour change of the samples resulting from the different proportions of water kefir and yeast was determined using a Konica Minolta CR-400 colourimeter (Tuscaloosa, NJ, USA). The films were placed on a white surface, and the CIELab colour space was utilised to determine the parameters L* (luminosity), a* (green to red) and b* (blue to yellow). The total colour change ∆*E* was calculated using Equation (2):

(2) $$\Delta E=\sqrt{{\left(L^{*}-L_{0}\right)}^{2}+{\left(a^{*}-a_{0}\right)}^{2}+{\left(b^{*}-b_{0}\right)}^{2}}$$

where *L*_0_, *a*_0_ and *b*_0_ are the coordinates corresponding to the sample 100K/0Y, considered the reference to determine the colour change. Five points were measured for each formulation.

#### 2.4.3. Microstructural Characterisation by Scanning Electron Microscopy (SEM)

The microstructure of films was analysed by observing the surfaces and cross sections using a scanning electron microscope FEI-Quanta 200 (Fei Co., Hillsboro, OR, USA) at 20 kV. Cross sections were obtained by cutting the samples with a sharp blade at room temperature. Subsequently, all samples were placed in the specimen holder and stored at 22 °C and 43% r.h. To enhance visibility under the microscope, the samples were coated with a layer of gold. Images of the surfaces (magnification 3000×) and cross sections (magnification 1500× and 20,000×) of the films were acquired under high-vacuum conditions.

#### 2.4.4. Attenuated Total Reflectance-Fourier Transform Infrared (ATR-FTIR) Spectroscopy Analyses

Infrared spectra of films were recorded in the range of 4000–500 cm^−1^ using a Fourier-Transform Infrared Analyser (FTIR) Shimadzu IR-Affinity (Shimadzu Co., Kyoto, Japan) equipped with an attenuated total reflectance diamond module (GladiATR, Pike Technologies, Madison, WI, USA). The spectra were obtained as an average of 48 scans with a resolution of 4.0 cm^−1^ and Happ–Genzel apodisation. A blank spectrum was obtained before each test to compensate for the effect of humidity and the presence of carbon dioxide in the air through spectra subtraction. The spectra were obtained in triplicate.

#### 2.4.5. Thermogravimetric Analyses (TGA)

To study the thermal degradation of the films, the weight loss of samples as a function of temperature was recorded using a TA Instruments Q-500 (New Castle, DE, USA) thermogravimetric analyser. The samples were equilibrated at 22 °C and 52% r.h. before the experiment. Approximately 6 mg of each sample was weighed in a platinum pan and heated from 30 to 800 °C at 10 °C min^−1^. The experiments were carried out in duplicate under a nitrogen atmosphere with a flow rate of 60 mL min^−1^. The initial degradation temperature (*T*_0_) was calculated as the temperature at which 10% of the weight was lost. The temperature at the maximum degradation rate (*T*_max_) was determined from the peak of the derivative of the weight loss with respect to temperature.

#### 2.4.6. Modulated-Temperature Differential Scanning Calorimetry (MDSC)

The thermal behaviour of films under controlled heating was studied using a MDSC (TA Instruments Q200, New Castle, DE, USA). Film samples were previously dehydrated at 22 °C in silica gel for 7 days. Approximately 5 mg of samples was placed into Tzero^®^ aluminium pans and sealed with hermetic lids. Thermograms were obtained in the range of −80 °C to 200 °C, with a previous equilibration step at −80 °C for 1 min. The temperature was increased at a rate of 10 °C min^−1^ with a modulation amplitude of ± 1 °C and a period of 40 s. Glass transition temperatures (*T*_g_) were determined from the reversible heat flow using TA Universal Analysis software (v4.5, TA Instruments, New Castle, DE, USA) at the midpoint. Experiments were performed in triplicate.

#### 2.4.7. Mechanical Uniaxial Tensile Tests

Uniaxial tensile tests of films were performed using a Universal Testing Machine (TC-500 II-Series, Micrometric, Argentina) equipped with a 300 N load cell. The samples were cut into rectangular shapes with a length of 50 mm and a width of 10 mm, and the effective distance between jaws was 25 mm. The tests were conducted at 22 °C, and film specimens were previously equilibrated at 52% of r.h. prior to testing. A testing speed of 10 mm min^−1^ was selected, and ten specimens of each composition were measured. The elongation at break *e*% (%), the maximum tensile strength *TS*_max_ (MPa) and Young’s modulus *Y* (MPa) were calculated from the resulting stress–strain curves as an average of ten measurements, following the ASTM D882 1997 standard [36].

#### 2.4.8. Water Sorption Isotherms

Water sorption isotherms were determined gravimetrically at 22 °C following the standard procedure previously described [6]. Dried samples of films with a surface area of 58 cm^2^ were placed in 3 L desiccators and equilibrated at different water activities (*a_w_*). Samples were periodically weighed using an analytical balance (±10^−4^ g), and the evolution of moisture content at each condition was monitored until a constant weight was achieved. The water content or hydration h, given in units of g of water per g of d.m., was evaluated as a function of *a_w_* (*a_w_* = % r.h./100). Experiments were performed in triplicate, and isotherms were fitted using the Guggenheim–Anderson–De Boer (GAB) model [37] through Equation (3):

(3) $$h\left(a_{w}\right)=\frac{Ncka_{w}}{\left[\left(1+\left(c-1\right)ka_{w}\right)\left(1-ka_{w}\right)\right]}$$

where *N* is the monolayer water content (g of water per g of d.m.) related to the primary binding sites of hydration water molecules; *c* is a parameter related to the sorption heat monolayer, which is associated with the force of the water binding to the monolayer; and *k* is a parameter related to sorption heat multilayer, which is linked to the capability of water to bind to the multilayer.

#### 2.4.9. Experimental Water Vapour Permeability Measurements

Experimental water vapour permeability (*P_w_^exp^*) of films was measured using the cup method described in ASTM-E96 2016 [38] with some modifications [30]. The films were sealed on the top of cups containing a saturated salt solution of BaCl_2_, which provides a high r.h. of 90%. Test cups were placed in 7 l desiccators maintained at a constant temperature of 22 °C and 10% r.h. A fan was used to maintain uniform conditions inside the desiccators over the films [39]. Weight loss measurements of the test cup, related to the water vapour transport across the film, were taken using an analytical balance (±10^−3^ g). Weight loss *m* (g) versus time *t* (min) was plotted, and when the steady state (a straight line) was reached, a further 36 h was recorded. *P_w_^exp^* was calculated as displayed in Equation (4):

(4) $$P_{w}{}^{exp}=\left(\frac{1}{A}\frac{\Delta m}{\Delta t}\right)\frac{L}{\Delta p_{w}}$$

where *A* = 2.2 ×10^−3^ m^2^ is the effective area of exposed film; Δ*m*/Δ*t* is the slope of a linear regression of the weight loss versus time; *L* (m) is the film thickness; Δ*p*_w_ = (*p*_w2_ − *p*_w1_) is the differential water vapour pressure across the film; and *p*_w2_ and *p*_w1_ are the partial pressures (Pa) of water vapour at the film surfaces inside and outside the cup, respectively [30]. *P_w_^exp^* is given in units of g s^−1^ m^−1^ Pa^−1^. Experiments were performed in triplicate.

### 2.5. Statistical Analyses

Statistical analyses were performed using OriginPro 8 (OriginLab Corporation, Northampton, MA, USA) and R software (v 3.4.4, R Foundation for Statistical Computing, Vienna, Austria). All results are shown as means with standard deviation. The data were subjected to the analysis of variance, and the means were compared by a post hoc test (Tukey HSD). Differences were considered significant at *p* < 0.05. Errors in the parameters from the Herschel–Bulkley and GAB models, obtained from the flow curves and water sorption isotherms, respectively, were estimated from the fit analysis.

## 3. Results and Discussion

### 3.1. Rotational Rheology of Film-Forming Dispersions

**Figure 1:**
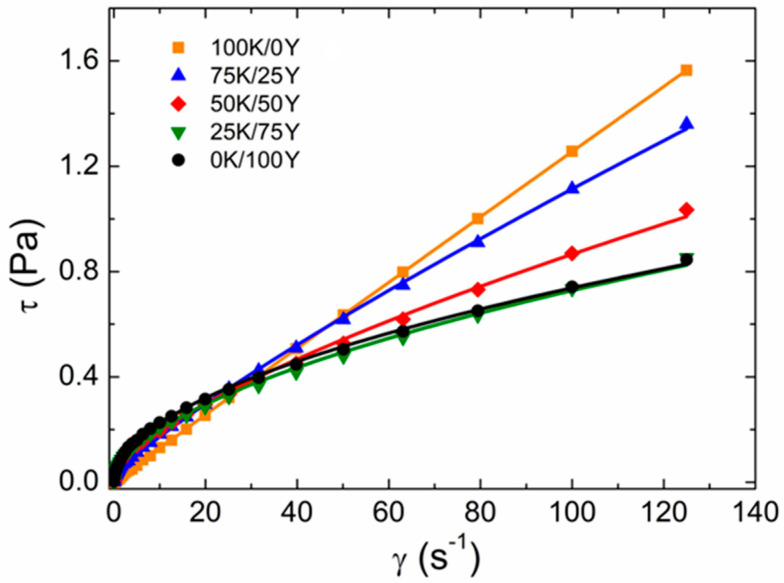
Rotational rheology of film-forming dispersions. Experimental data were fitted with the Herschel–Bulkley model (Equation (1)). Fitted parameters are shown in Table 1.

All film-forming dispersions obtained from blends of water kefir grains and yeast were homogeneous without further phase separation. The flow behaviour of the dispersions can be seen in Figure 1. The experimental points of the shear stress *τ* as a function of the deformation rate *γ* were fitted with the Herschel–Bulkley model (Equation (1)), and the parameters obtained from the fit are displayed in Table 1. The reported values of the statistical parameter *R*^2^ indicate a good fit.

All dispersions exhibited non-Newtonian rheological behaviour after reaching the yield stress *τ*_0_. Moreover, as shown in Table 1, all dispersions behaved as pseudoplastic fluids (*η* < 1). The results suggested that as the yeast content in the dispersion increased, its pseudoplastic performance became more prominent, as the pseudoplastic behaviour of a dispersion is more pronounced when the flow index *n* is lower. On the other hand, the consistency index *K* monotonically increased as a function of the yeast content in the dispersion from (14 ± 1) × 10^−3^ Pa s for sample 100K/0Y to (67 ± 2) × 10^−3^ Pa s for sample 0K/100Y. These results indicated that the apparent viscosity notably increased as the amount of yeast in the dispersion augmented. Therefore, the incorporation of water kefir grains could provide a processing advantage during high-shear processing operations.

It could be observed in Table 1 that *τ*_0_ presented lower values for samples 100K/0Y and 0K/100Y, while the blends displayed higher values of τ0 as compared to the single biomass dispersions. Fluids with values of *τ*_0_ > 0 behave as solids until they exceed the yield stress; then, for values of *τ*> *τ*_0_, they behave like liquids. Higher values of *τ*_0_ were caused by the interaction between the dispersed components [40,41]. This suggested that the thermal treatment performed on the dispersions exposed the hydroxyl groups of kefir dextrans and the amino groups of the yeast proteins to direct interactions, leading to the formation of intermolecular hydrogen bonds between the two components and increasing *τ*_0_. These results showed the existence of interactions between water kefir grains and yeast biomasses.

### 3.2. Quality Evaluation, Visual Appearance and Microstructure of Films

As a visual assessment, all the films were smooth with a uniform surface, free of air bubbles, cracks and pores, and had good handleability and continuity. However, the 25K/75Y samples showed some roughness and were less homogeneous. The 100K/0Y samples were transparent and very flexible, as was reported previously [28]. Increasing yeast content led to decreased flexibility and an increase in yellow-brown colour, but all samples remained translucent. The films had a uniform thickness of 0.13 ± 0.01 mm.

**Figure 2:**
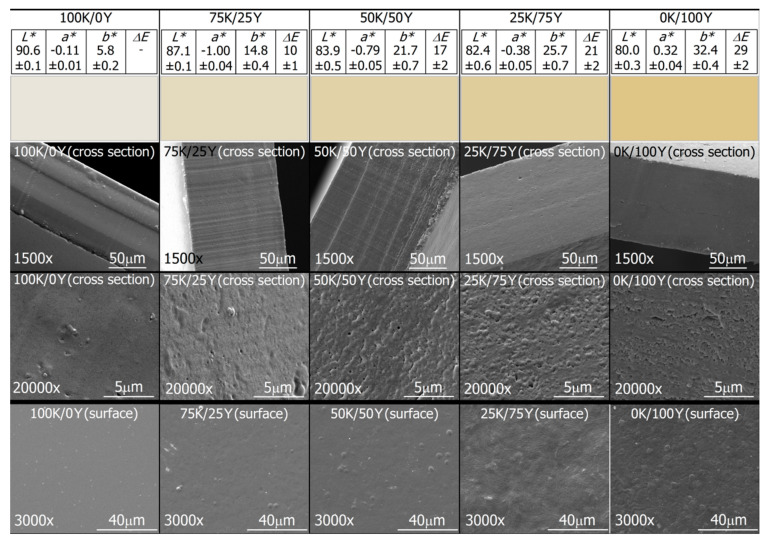
Colour appearance and SEM micrographs of films. Row 1: CIELab coordinates and colour representation of films. Rows 2, 3 and 4: Cross section at 1500×, cross section at 20,000× and surface of the films at 3000×, respectively.

The colour of packaging materials is important, as it affects the appearance of the product. Transparent films allow the product inside to be visible, while coloured films can provide protection from radiation. The CIELab colour parameters and colour representation of the films are shown in Figure 2, Row 1. The sample 100K/0Y was used as the standard to calculate Δ*E* due to its natural transparency. The L* values were high in all films, particularly for the 100K/0L sample, indicating high clarity and transparency. However, the L* values decreased with the addition of yeast biomass. As yeast content increased, a slight trend towards red colour (parameter a*) and yellow (parameter b*) was observed due to Maillard reactions between yeast proteins and carbohydrates. The Δ*E* value was greater than 6 for all samples, indicating that the colour difference between the films is noticeable to the human eye. These results were consistent with visual observations.

Microstructural analysis based on SEM micrographs was conducted to evaluate the homogeneity of the films and compatibility between different constituents (Figure 2, Rows 2–4). Increasing yeast content resulted in different morphologies of the micrographs. The 100K/0Y sample exhibited a homogeneous and continuous structure without any agglomerations, defects or perforations, indicating the formation of a single compact phase. The 0K/100Y sample had a structure without fractures but was less homogeneous than the 100K/0Y sample. Scattered ovoid structures were detected on the surface images of the 0K/100Y film, which were attributed to remnants of yeast cell walls that retained their shape after physical treatments. The hybrid samples 50K/50Y and 75K/25Y did not generate any additional phase. However, a slight microphase separation was observed in the 25K/75Y sample, suggesting partial integration between the two biomasses.

These findings demonstrate that the applied physical treatments successfully disintegrated the granular and cellular structures in the water kefir grain and yeast biomasses. This allowed the release of the protein-rich cytoplasmic content and the unfolding of biopolymers, promoting the interleaving and formation of a polymer network that contributed to the production of uniform films, without any discernible phase separation between the components. Interactions between the blend components were evident in all cases, resulting in a continuous matrix.

### 3.3. Fourier Transform Infrared (ATR-FTIR) Spectroscopy of Films

**Figure 3:**
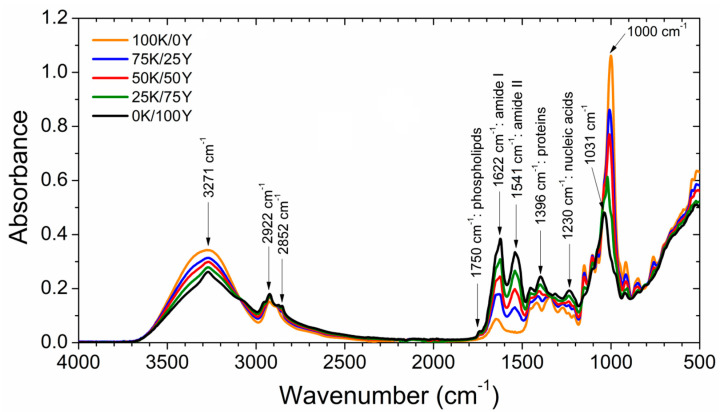
FTIR spectra of films.

As can be seen in Figure 3, the proportion of biomasses in each blend influenced the FTIR spectra of the films. There are characteristic bands present in the spectra, which can be attributed to the components of water kefir grains and yeast biomasses. The range between 1200 and 1500 cm^−1^ exhibited changes due to the presence of yeast biomass. For instance, the bands associated with proteins (1396 cm^−1^) and nucleic acids (1230 cm^−1^) were greatly affected by the incorporation of yeast biomass. The complete FTIR spectra of the films can be seen in Figure 3, which allows for a region-by-region analysis.

The peak at 3271 cm^−1^, associated with the absorption of hydroxyl groups (-OH) of water and polysaccharides, was most intense in the range between 3600 and 3000 cm^−1^ [28]. As the yeast content increased, a decrease in absorbance was observed in this band, and its shape changed due to the lower carbohydrate content of yeast as compared to water kefir. The binding of the C-H bond of the methyl and methylene functional groups was observed in the range of 3000–2800 cm^−1^, with similar intensity in all films.

In the mid-region, a peak at 1750 cm^−1^ corresponding to yeast phospholipids was detected [27], which decreased with the addition of kefir. The zone between 1700 and 1380 cm^−1^ showed peaks at 1622, 1541 and 1396 cm^−1^, attributed to amide groups [27]. These bands lost intensity and shifted as the kefir content increased, as expected due to the very low quantity or absence of proteins in water kefir grains [28]. However, a peak near 1630 cm^−1^ was evident in the 100K/0Y sample, which could be associated with the balancing vibration of the -OH group of the water molecules [42].

In the range between 1390 and 1190 cm^−1^, peaks that changed both shape and position were observed, indicating interactions between the biomass constituents of the blend. Moving on to the range of 1200–900 cm^−1^, two major peaks were detected at 1031 and 1000 cm^−1^, which were associated with yeast mannans and glucans [27] and water kefir dextrans [28], respectively. Additionally, secondary peaks attributed to carbohydrate ring vibrations and secondary groups (C-O-C; C-OH; C-H) were also detected in this region, and their intensity decreased with an increase in yeast content in the films. These peaks may be attributed to the vibrational movements of the glucose units, which constitute the dextran present in water kefir grains [28].

### 3.4. Thermogravimetric Analysis of Films

**Figure 4:**
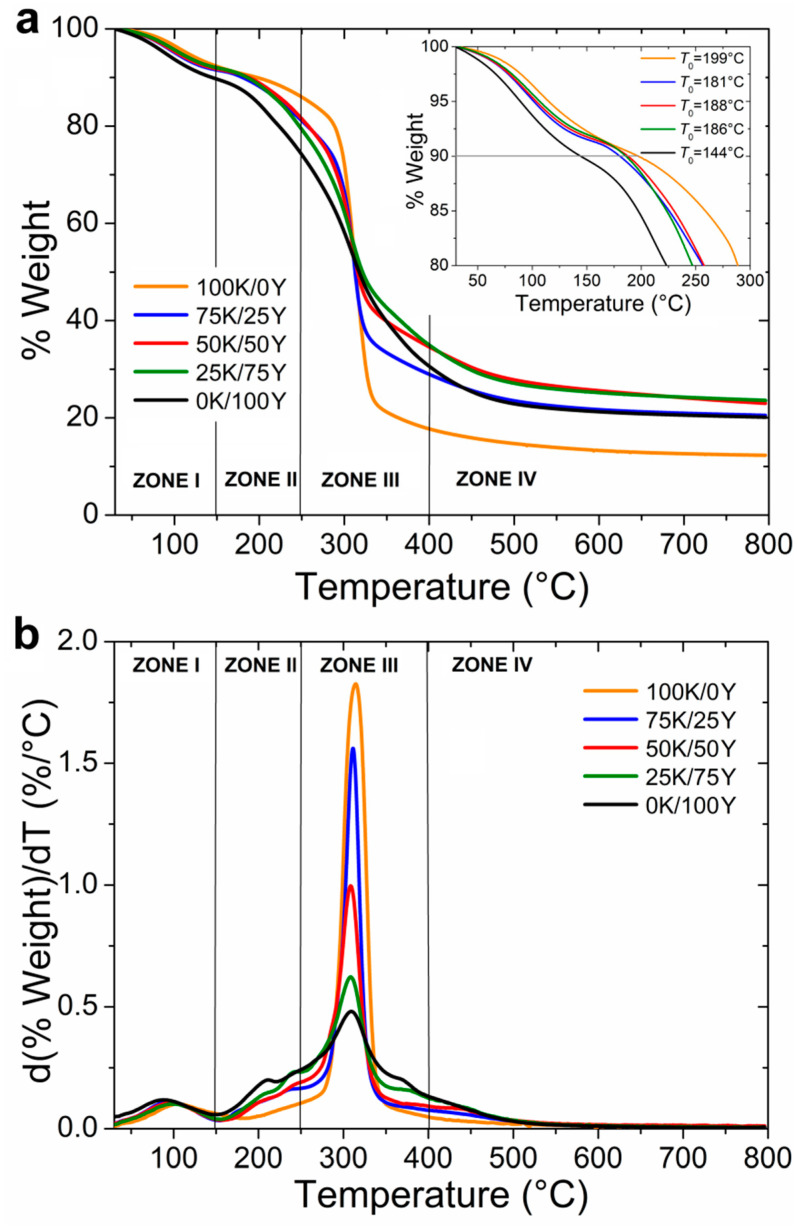
Thermogravimetric analyses of films. (**a**) Weight loss of films previously hydrated at 53% r.h. The initial degradation temperatures *T*_0_ are displayed in the zoom. (**b**) Derivative of weight loss of films with respect to the temperature.

The thermogravimetric analysis of the hydrated films at 52% r.h. is presented in Figure 4. As shown in Figure 4a,b, four distinct thermal degradation zones were observed.

The first zone, which occurred below 150 °C, was attributed to dehydration and possible volatilisation of low molecular weight compounds [6,43]. The weight loss in this region was approximately 10%. Figure 4a clearly indicates that the thermal stability of the films increased with the incorporation of water kefir into the blend. This was also evident in the T_0_ values, which increased with the addition of water kefir to the material.

A second thermal degradation event could be identified in the range of 150–250 °C, which was more significant in the 0K/100Y sample and showed a decrease in intensity as water kefir content increased. This zone could be related to glycerol degradation and the onset of yeast proteins’ decomposition [27], explaining why thermal degradation in this temperature range decreased with the increasing amount of water kefir in the sample.

The third degradation zone, between 250 °C and 350 °C, presented the maximum degradation rate of the samples. The T_max_ values obtained from Figure 4b for the 100K/0Y, 75K/25Y samples were 315 and 311 °C, respectively, while the value obtained for the 50K/50Y, 25K/75Y and 0K/100Y samples was 309 °C. In this region, the 100K/0Y film showed a higher weight loss of around 69%, while the 0K/100Y sample presented a lower weight loss of around 45%. These results corresponded to those reported for polysaccharides [44] and protein degradation [45]. Thus, this region could be attributed to the degradation of dextrans of water kefir, mannans and glucans of yeast, and the complete degradation of proteins. Figure 4b showed that as the proportion of yeast increased, there was a noticeable attenuation of the peak. This effect was mainly due to the lower polysaccharide content of the yeast and the fact that some proteins were previously degraded in Zone II. The sharp peak presented in Figure 4b for the 100K/0Y sample described that those materials were mainly composed of one component (dextran), whereas a spread peak was observed in 0K/100Y films due to the complex composition of these films.

Finally, a weight loss was observed between 400 °C and 800 °C, with a remaining residue of approximately 12% to 24%. As shown in Figure 4a, the film with 100% water kefir had the highest total weight loss, followed by the one with 100% yeast. This finding is significant, as it indicates that the combination of water kefir and yeast biomasses, particularly the 50K/50Y and 25K/75Y ratios, generated stronger interactions with higher thermoresistance than their pure components isolated. This interaction effect between the water kefir and yeast biopolymers was also evident in the rheological tests of the dispersions discussed in Section 3.1, as well as in FTIR analysis results.

### 3.5. Differential Scanning Calorimetry and Glass Transition Temperature of Films 

**Figure 5:**
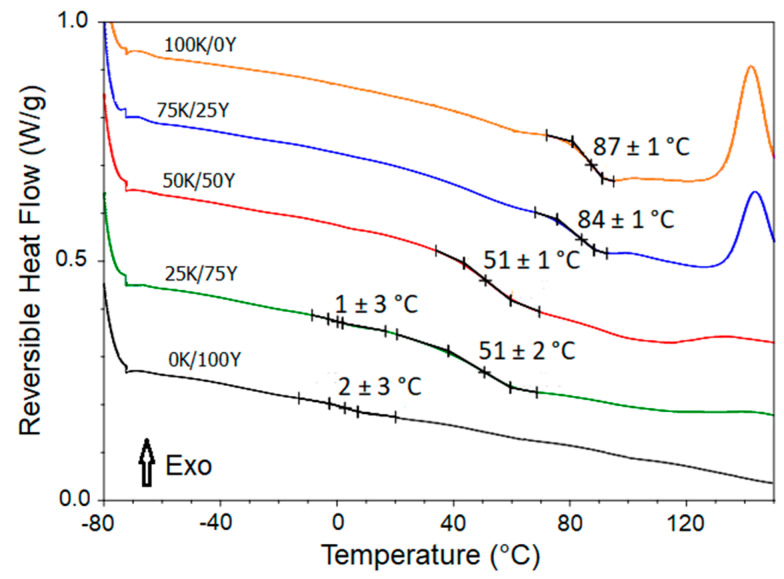
DSC thermograms and glass transition temperature of dried films.

The primary method for investigating the number of amorphous phases that exist in a polymer blend material is by determining the number of glass transition temperatures, as each T_g_ corresponds to one amorphous phase [10]. However, if only one T_g_ is detected, this could suggest complete miscibility, which should be further confirmed. Figure 5 displays the thermograms of the reversible heat flow for the previously dehydrated films.

According to Figure 5, the 100K/0Y film had the highest *T*_g_ at 87 ± 1 °C. Upon incorporating 25 % of yeast biomass (sample 75K/25Y), a slight decrease in *T*_g_ was observed to 84 ± 1 °C. For the 50K/50Y sample, the measured *T*_g_ was 51 ± 1 °C. At this biomass ratio, the interaction between the different components of the material and the plasticising effect decreased the *T*_g_ value. In the 25K/75Y sample, two *T*_g_ values were observed, one at 51 ± 2 °C and the other at 1 ± 3 °C. The 0K/100Y film presented a *T*_g_ at 2 ± 3 °C, confirming the plasticisation of the material by components of the yeast biomass.

Previous studies have reported that the presence of two *T*_g_ in a material indicates the existence of two different phases [46,47]. Therefore, the observation of two *T*_g_ values in the 25K/75Y sample suggested a partial phase separation from a calorimetric perspective. These findings were consistent with the results obtained from visual inspection and micrographs of this sample, which showed roughness and inhomogeneity (refer to Section 3.2).

### 3.6. Mechanical Properties of Films

**Figure 6:**
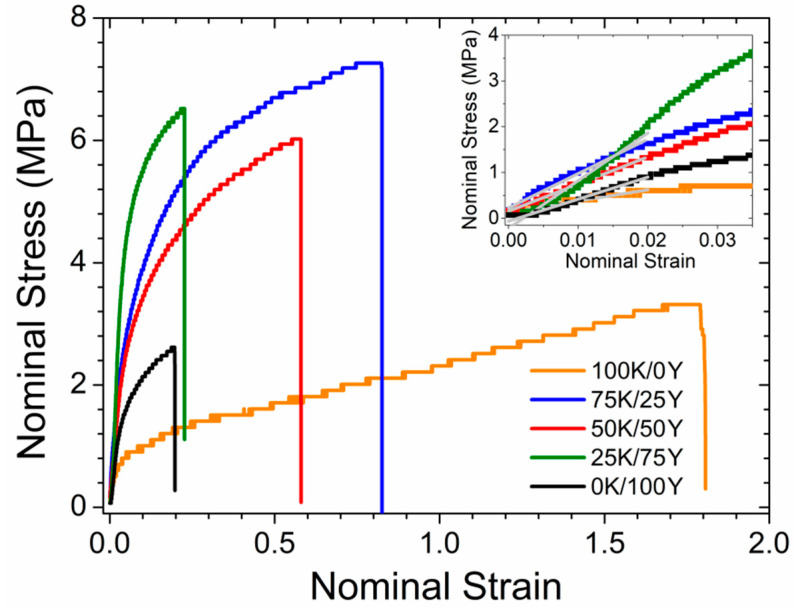
Representative stress–strain curves for one of ten replications of the mechanical test performed for films at 52 % r.h. Y was calculated from the slope in the linear region (insert of the figure), *TS*_max_ from the maximum value of the nominal stress and *e*% from the maximum value of the nominal strain. Values of the mechanical parameters are shown in Table 2.

The mechanical properties of a material depend on its composition, structure and interactions between the constituent polymers. Young’s modulus (*Y*), maximum tensile strength (*TS*_max_) and elongation at break (*e*%) were calculated from stress–strain curves (Figure 6) and presented in Table 2.

Table 2 shows that *e*% increased significantly as the content of water kefir biomass in the samples augmented. Water kefir-based films plasticised with glycerol exhibit a remarkable characteristic of high elongation capacity as compared to some synthetic materials, such as high-density polyethylene (150 ± 8%), and other biopolymeric films reported in the literature [28]. The results indicated that the incorporation of water kefir biomass improved the elongation capacity of yeast-based films.

On the other hand, *TS*_max_ increased with the increasing amount of yeast in the films, resulting in improved strength. However, this value was the lowest for films based on yeast biomass alone. Films composed of water kefir and yeast biomasses showed a synergistic behaviour in terms of this value, probably due to a high number of interpolymer bonds, implying an increase in *TS*_max_ compared to films based on a single biomass. This effect on TS_max_ was also observed in films based on polymeric blends of starch/chitosan [17,21] and kefiran/starch [24], but it was not detected in films based on blends of water kefir grains biomass/chitosan [26].

The *Y* parameter showed a similar dependence as the *TS*_max_ had for the different proportions of kefir and yeast, with the lowest values observed in films based on single biomass and an increase in samples containing both biomasses. This finding suggested that the combination of water kefir and yeast biomass generated stronger interactions, leading to an increase in both *Y* and *TS*_max_. These results were consistent with the findings from the thermogravimetric analyses of the films (see Section 3.4).

Furthermore, the results indicated differences in the structure and interpolymeric bonds for the different proportions of water kefir and yeast. Similar behaviour of mechanical parameters *Y* and *TS*_max_ was also observed in films based on polymeric blends of kefiran and starch, suggesting the presence of complex interactions [24].

### 3.7. Hydration and Water Vapour Permeability of the Films

**Figure 7:**
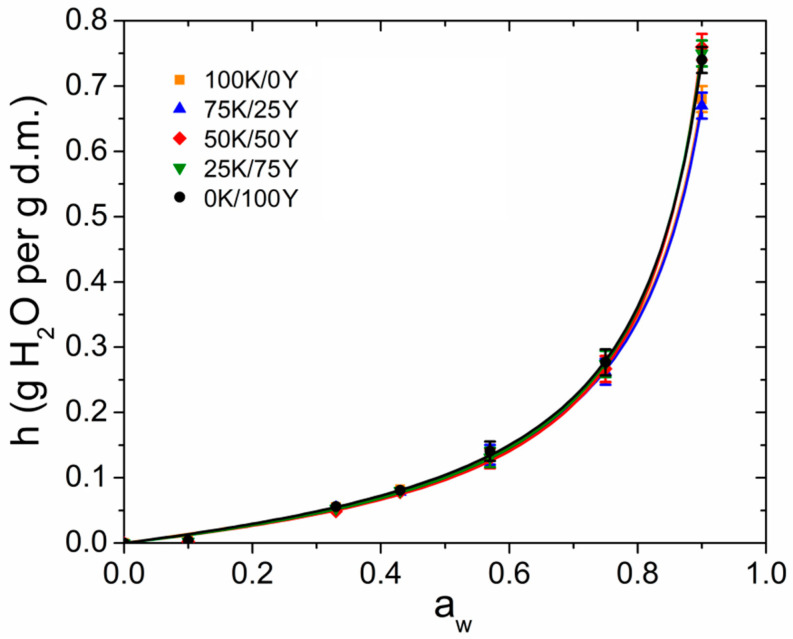
Water vapour sorption isotherms of films. Experimental data were fitted with the GAB model using Equation (3). The fitting parameters are shown in Table 3.

Biopolymer-based films are hydrophilic biodegradable matrices, which interact strongly with water and exhibit a poor water vapour barrier. Therefore, it is crucial to study their hydration properties [48]. Understanding these properties is essential for predicting the optimal storage conditions, shelf life and behaviour of these materials under different humidity conditions. Water sorption isotherms provide valuable information about the interaction between water and the film matrix, as well as on the structural arrangement of water within the material. Figure 7 presents the water sorption isotherms for different mixing biomass ratios. The experimental data were fitted using the GAB model, represented by Equation (3). The parameters obtained from the fit are presented in Table 3.

As shown in Figure 7, there was little variation in the isotherms of films with different proportions of kefir and yeast. An increase in yeast content led to higher hydration equilibrium values at high relative humidity (*h*_90% r.h._*)* (see also Table 3). The isotherms displayed a slight increase in the hydration water content at low *a*_w_ values, followed by a sharp increase for *a*_w_ > 0.6. This indicates that most of the hydration water molecules were arranged forming multilayers, while a small amount of water was directly bound to the polymeric matrix, forming the hydration monolayer. This water sorption behaviour is commonly observed in hydrophilic materials made from biopolymers [30,49], suggesting that hydration water molecules in biopolymeric films can be transported through a sorption–diffusion–desorption mechanism driven by a water vapour pressure gradient [30].

As shown in Table 3, the parameters *N* and *c* characterising the monolayer (see Section 2.4.8) did not show substantial differences among the different proportions of water kefir and yeast. However, the parameter *k* (as well *h*_90% r.h._) increased with the addition of yeast biomass in the samples. This suggested that the observed differences in hydration were mainly due to the changes in the water content in the multilayer.

The water vapour permeability of biopolymer-based films is an important property, which indicates their ability to control water vapour transport between a system, e.g., food, and its surroundings [50]. Microstructural studies revealed that all films had a continuous microstructure without cracks (see Section 3.2). Consequently, water transport in these films occurs not through pores but via the sorption–diffusion–desorption mechanism [30]. In biopolymeric films, the water vapour permeability is strongly dependent on the experimental conditions, including water pressure gradient and film thickness [30]. Therefore, in order to compare the *P_w_^exp^* values of different films, they must have the same thickness (0.13 ± 0.01 mm in our case) and identical conditions of the water pressure gradient. Table 3 displays the values of *P_w_^exp^* measured for the films with different proportions of water kefir and yeast. It was observed that *P_w_^exp^* ranged from 5.2 ± 0.1 to 6.2 ± 0.1 × 10^−10^ g m^-1^ s^−1^ Pa^−1^. These values do not indicate a large change in *P_w_^exp^* due to the different proportions of kefir and yeast. As could be observed in Table 3, a clear trend could not be established regarding the performance of *P_w_^exp^* as a function of the components of the blends, indicating complex interactions. Similar behaviour has been reported in the study of the permeability of chitosan–starch polymer blends [15].

## 4. Conclusions

Blending yeast biomass and water kefir grain biomass to develop new biodegradable films presented a synergistic effect, which improved certain characteristics of the films compared to using each biomass individually. The films obtained from blending the entire biomasses of the water kefir grains and yeast displayed excellent continuity and homogeneity without any cracks. The amount of water kefir grain biomass in the formulation had a significant impact on increasing the clarity, transparency, as well as the elongation at break of the films.

Infrared spectroscopy, thermal degradation analyses and mechanical properties showed that the combination of water kefir and yeast biomasses generated stronger interactions, enhancing thermomechanical functional properties compared to films based on a single biomass. 

Since the applicability of these films depends on their functional properties, determining the optimal ratio of biomasses involves considering all of the characteristic properties depending on the required application.

The mixture of both biomasses proved to be compatible, and their integral use to prepare films could be advantageous due to the cost effectiveness of the process (no separation or purification), their interesting physical–chemical properties and the retention of natural bioactive substances. The materials obtained in this work presented promising characteristics to be used as biodegradable packaging.

## Figures and Tables

**Table 1:** Values of the parameters obtained from fitting the Herschel–Bulkley model (Equation (1)) to the flow curves of Figure 1. Errors in the parameters were estimated from the fit analysis.

Sample	*τ*_0_ (10^−3^ Pa)	*K* (10^−3^ Pa.s)	*n*	*R* ^2^
100K/0Y	1.7 ± 0.3	14 ± 1	0.98 ± 0.01	0.999
75K/25Y	16 ± 2	22 ± 1	0.85 ± 0.01	0.999
50K/50Y	31 ± 2	32 ± 2	0.71 ± 0.01	0.998
25K/75Y	34 ± 2	54 ± 2	0.56 ± 0.01	0.997
0K/100Y	2 ± 1	67 ± 2	0.52 ± 0.01	0.999

**Table 2:** Mechanical parameters of films. The mean and standard deviation (*n* = 10) are reported. The different letters assigned in each column refer to significant differences (*p* ≤ 0.05).

Sample	*Y* [MPa]	*TS*_max_ [MPa]	*e*% (%)
100K/0Y	34 ± 5 ^e^	3.8 ± 0.5 ^c^	180 ± 20 ^a^
75K/25Y	75 ± 7 ^b^	7.0 ± 0.4 ^a^	82 ± 9 ^b^
50K/50Y	56 ± 5 ^c^	6.2 ± 0.3 ^b^	57 ± 4 ^c^
25K/75Y	118 ± 11 ^a^	6.6 ± 0.3 ^ab^	22 ± 2 ^c^
0K/100Y	44 ± 4 ^d^	2.6 ± 0.2 ^d^	20 ± 2 ^c^

**Table 3:** Values of the GAB parameters fitted for the water sorption isotherms displayed in Figure 7 and experimental water vapour permeability *P_w_^exp^* (10^−10^ g s^−1^ m^−1^Pa^−1^) of films. Errors in the GAB parameters were estimated from the fit analysis. *h*_90% r.h._ refers to hydration equilibrium values measured at 90% r.h. The different letters assigned in each column refer to significant differences (*p* ≤ 0.05).

Samples	*h*_90% r.h._ (g/g)	GAB Parameters				*P*_w_^exp^
		*N* (g/g)	*c*	*k*	*R* ^2^	
100K/0Y	0.68 ± 0.02 ^b^	0.089 ± 0.008	1.52 ± 0.35	0.973 ± 0.008	0.999	5.2 ± 0.1 ^a^
75K/25Y	0.67 ± 0.02 ^b^	0.089 ± 0.008	1.46 ± 0.35	0.974 ± 0.009	0.999	6.0 ± 0.1 ^c^
50K/50Y	0.76 ± 0.02 ^a^	0.085 ± 0.007	1.33 ± 0.31	0.996 ± 0.007	0.999	5.3 ± 0.1 ^ab^
25K/75Y	0.75 ± 0.02 ^a^	0.090 ± 0.008	1.30 ± 0.30	0.992 ± 0.008	0.999	5.5 ± 0.1 ^b^
0K/100Y	0.74 ± 0.02 ^a^	0.091 ± 0.007	1.44 ± 0.33	0.990 ± 0.008	0.999	6.2 ± 0.1 ^c^

## Data Availability

The data presented in this study are available on request from the corresponding author.
